# A tent-like sign during endoscopic ultrasound-guided gastroenterostomy: an indication of a misdeployed stent in the peritoneum

**DOI:** 10.1055/a-2106-1544

**Published:** 2023-07-27

**Authors:** Yu-Ting Kuo, Hsiu-Po Wang

**Affiliations:** 1 Division of Endoscopy, Department of Integrated Diagnostics and Therapeutics, National Taiwan University Hospital, Taipei, Taiwan; 2 Department of Internal Medicine, College of Medicine, National Taiwan University, Taipei, Taiwan; 3 Department of Internal Medicine, National Taiwan University Hospital, Taipei, Taiwan

Endoscopic ultrasound-guided gastroenterostomy (EUS-GE) with the use of a lumen-apposing metal stent (LAMS) is an established alternative technique for patients with malignant gastric outlet obstruction 1 . Although single-step delivery systems for LAMSs significantly reduce the risk of adverse events (AEs), stent misdeployment during EUS-GE remains the most common cause for technical failure and AEs. This report describes the case of a patient who underwent EUS-GE with deployment of the distal flange of the LAMS occurring in the peritoneum (type 1 stent misdeployment) 2 .

A 57-year-old man with advanced pancreatic cancer with multiple liver metastases attended our hospital because of early satiety and post-prandial vomiting for several weeks. After admission, he underwent esophagogastroduodenoscopy, which confirmed pancreatic cancer with duodenal bulb invasion. EUS-GE with the direct technique was performed using a 20-mm cautery-enhanced LAMS (Hot AXIOS; Boston Scientific, Marlborough, Massachusetts, USA). After a suitable location for a gastroenterostomy had been identified, direct puncture with the LAMS was performed from the gastric wall into the target jejunum; however, the tip of the delivery system of the LAMS caused the bowel wall to deform into a shape that produced a tent-like sign owing to incomplete penetration of the bowel wall. This sign warrants caution as it indicates misdeployment of the distal flange of the LAMS in the peritoneum ( Fig. 1 , Fig. 2 ; Video 1 ).

**Fig. 1:**
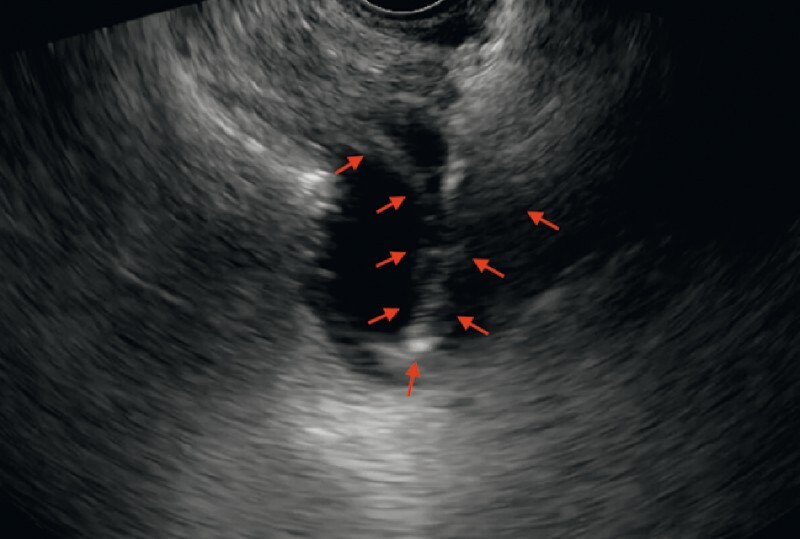
Endoscopic ultrasound (EUS) image showing the tent-like sign of the bowel wall (arrow) that resulted from the tip of the delivery system of the lumen-apposing metal stent incompletely penetrating the targeted jejunum during EUS-guided gastroenterostomy.

**Fig. 2:**
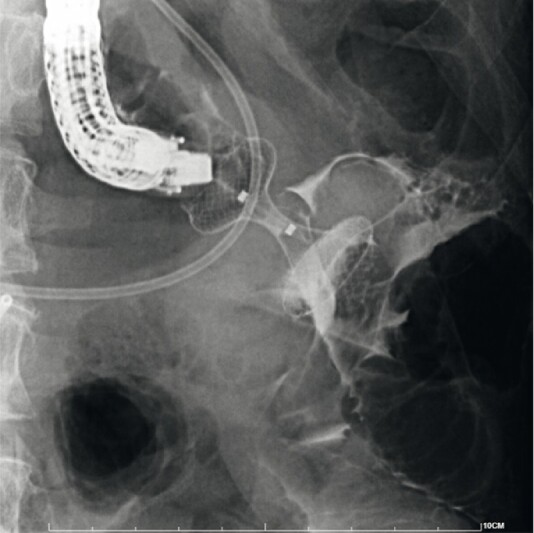
Fluoroscopic image showing the misdeployment of the lumen-apposing metal stent, with the distal flange seen, after contrast injection, in the peritoneum and the proximal flange in the stomach.

**Video 1** An attempted endoscopic ultrasound-guided gastroenterostomy results in misdeployment of the distal flange of a lumen-apposing metal stent in the peritoneum, which was managed by removal of the misdeployed stent and endoscopic closure of the subsequent gastrotomy using through-the-scope clips. A second attempt during the session proved successful.


The complication was managed by removal of the misdeployed LAMS followed by endoscopic closure of the gastrotomy using through-the-scope (TTS) clips ( Fig. 3 ). A subsequent EUS-GE using a second new 20-mm cautery-enhanced LAMS was performed successfully during the same session. No obvious pneumoperitoneum was observed after these procedures ( Fig. 4 ) and the patient’s symptoms subsequently resolved.

**Fig. 3:**
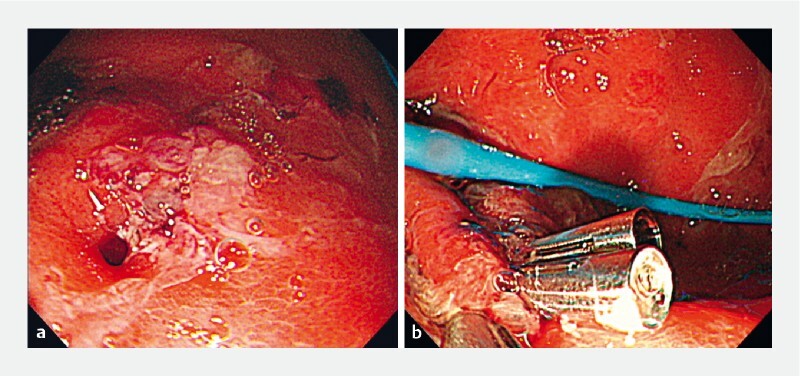
Endoscopic images showing: **a** the gastrotomy left after removal of the misdeployed lumen-apposing metal stent; **b** through-the-scope clips placed to close the gastrotomy endoscopically.

**Fig. 4:**
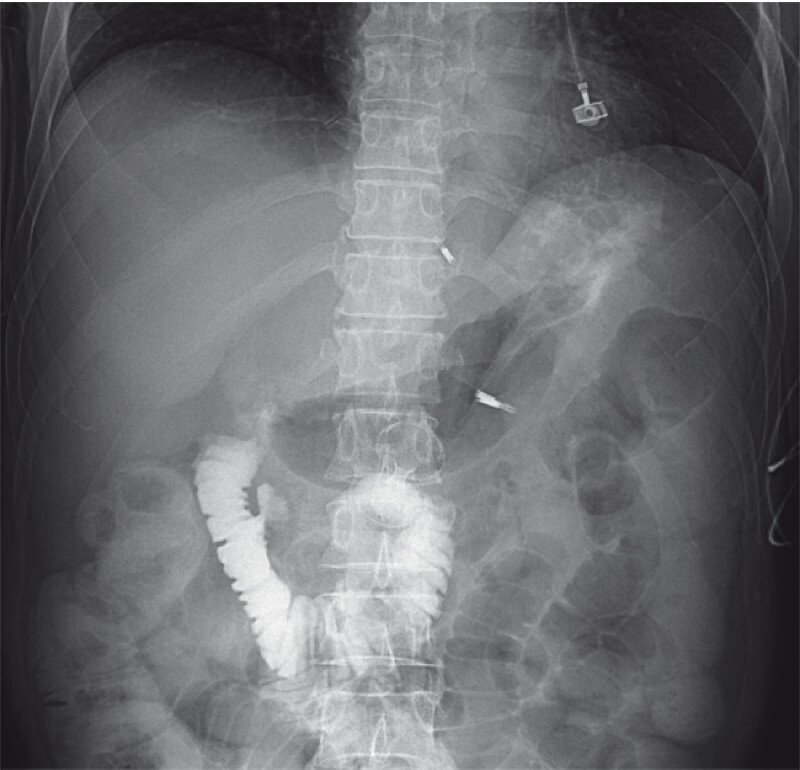
Radiographic image after a second endoscopic ultrasound-guided gastroenterostomy, using a new 20-mm cautery-enhanced lumen-apposing metal stent, had been successfully performed in the same session showing no obvious pneumoperitoneum.

Caution should be taken in the event of the rare and dangerous tent-like sign, which indicates incomplete puncture through the bowel wall, during EUS-GE with LAMS deployment.

Endoscopy_UCTN_Code_TTT_1AS_2AG

## Notes

Endoscopy E-Videos

https://eref.thieme.de/e-videos
*E-Videos* is an open access online section of the journal *Endoscopy* , reporting on interesting cases and new techniques in gastroenterological endoscopy. All papers include a high-quality video and are published with a Creative Commons CC-BY license. Endoscopy E-Videos qualify for HINARI discounts and waivers and eligibility is automatically checked during the submission process. We grant 100% waivers to articles whose corresponding authors are based in Group A countries and 50% waivers to those who are based in Group B countries as classified by Research4Life (see: https://www.research4life.org/access/eligibility/ ). This section has its own submission website at https://mc.manuscriptcentral.com/e-videos
